# Supplementary material for: Phylogenetic and evolutionary analysis of dengue virus serotypes circulating at the Colombian–Venezuelan border during 2015–2016 and 2018–2019
Source: PLoS One. 2021 May 28;16(5):e0252379. doi: 10.1371/journal.pone.0252379 (PMC8162668; doi:10.1371/journal.pone.0252379)
Supplement: Attachment — Submitted filename: Response to reviewers (05-05-2021).docx [file pone.0252379.s003.docx]

**RESPONSE TO REVIEWERS COMMENTS (PONE-D-20-27563R1)**

Phylogenetic and evolutionary analysis of Dengue Virus serotypes circulating at the Colombian–Venezuelan border during 2015–2019

Marlen Yelitza Carrillo-Hernández, Julian Ruiz-Saenz, Lucy Jaimes Villamizar, Sara Maria Robledo Restrepo, Marlen Martínez-Gutierrez

**Additional Editor Comments**

1. In this study, the authors collected the clinical samples during 2015-2016 and 2018-2019. The use of 2015–2019 in the Title was inappropriate.

**Answer from author:**

We agree with the editor comment and modify the title according to the recommendation (Lines 1-2)

2. The sample size of 229 suspected DENV samples was relatively small. In particular, of 41 samples positive for DENV, only 10 E gene sequences were obtained. Why so few sequences to be obtained? The proportion of successfully sequencing was substantially lower than a previous report (J Travel Med. 2020, 27(7): taaa195). Could the authors use RT-nested PCR to amplify the E gene (Emerg Infect Dis. 2012, 18(11):1850-7) ? Too few sequences do not result in a solid result or conclusion. This point should be mentioned as a limitation.

**Answer from author:**

We agree to the reviewer; the low number could be a Limitation. However, it is clear that our research area has a different epidemiological panorama in comparison to the one described by Ma et al., in Shanghai (J Travel Med. 2020, 27(7): taaa195). In the paper mentioned by the reviewer, most of the DENV cases were imported from Southeast Asian countries (aprox 60%). In the studied Colombian–Venezuelan border we have published that although patients usually are described as DENV, most of the cases could be other viruses such as Chikungunya, Zika or other unidentified pathogens (Carrillo et al., doi: 10.1186/s12879-018-2976-1). Therefore, to clarify this point, a short sentence about this topic was added to the Discussion (line 501-504). Besides, One Step-rtPCR was used to obtain ENV sequences in low RNA quality and problematic Samples. This was also better explained in the Methods Section (Line 138-144).

3. The molecular epidemiology of four DENV serotypes in America (especially in Venezuela and Colombia) should be mentioned in Introduction. If four DENV serotypes are co-circulating in a continent/region, cross-border transmission of all four serotypes were often observed among neighboring countries. The compelling evidences are from Asia, where all four DENV serotypes were introduced into China from neighboring countries along land border ports (e.g. China-Myanmar border), and/or surrounding countries via air travel (e.g. between Shanghai, China and some Southeastern Asian countries) (Emerg Infect Dis 2018; 24:1756–8，J Travel Med. 2020, 27: taaa195). If all four serotypes co-circulating in America, why no DENV-3 and DENV-4 to be detected? And also, no cross-border transmission of DENV-3 and DENV-4 in these regions? This point should be mentioned and discussed (maybe as a limitation) in the paper.

**Answer from author:**

We thank the editor for the recommendation and included a short paragraph in the Introduction to explain the molecular epidemiology of DENV in Venezuela (Line 67-71). Also, we modified the Discussion to clarifies the information (Line 504-508).

3. the cross-border transmission of all four serotypes were observed between

**Answer from author:**

Unfortunately, we do not understand the editor comment.

4. The structure of the Results should be reorganized. Too many sections are included in Results. Phylogenetic analysis, Phylogenetic analysis of DENV-1, and Phylogenetic analysis of DENV-2 should be merged into one section (e.g. Phylogenetic analysis). Similarly, Evolutionary analysis, DENV-1 E gene phylogeography and DENV-2 E gene phylogeography should be merged into one section of “Phylogeographic analyses”.

**Answer from author:**

We agree to the editor. The results were modified according to the recommendation to a better understanding of the information.

5. In the Phylogenetic analyses of DENV-1 and -2, it makes no sense to further divide genotypes into clades and even sub-clades. I suggest to remove these classification.

**Answer from author:**

We partially disagree to the reviewer. Although it has been widely discussed the use of Genotypes subclassifications into Clades and subclades; in our paper, this classification allows us to understand inter-genotype and intra-genotype viral genome diversity. Besides, it allows to achieve a full comparison to other previously published papers in the region of the Americas. We support the use of this subclassification with some references in the Discussion section (lines 422-428)

6. In the phylogeographic analysis of DENV-1, first introduction should not be traced back to 1973 (it should be later than 1973, maybe about 1980). In addition, the identification of second, third and fourth introductions was incorrect. The so-called three introduction events were more likely to be a single introduction event with a tMRCA in around 1994 (at the next node of the 1993 node).

**Answer from author:**

We agree to the reviewer and apologize for the involuntary mistake in the presented data for DENV-1. We developed a new phylogeographic analysis for DENV-1 by including missing sequences. The full data and trees fully agree to the reviewer corrections and were included in the new version of the manuscript (Lines 285-288 and 290-320) and a new figure 4 was included too.

7. In the phylogeographic analysis of DENV-2, there might be two independent introduction events of DENV-2 from Venezuela to Colombia in around 1998 and 2001. Since then, frequent cross-border transmissions of DENV-2 between Venezuela and Colombia were observed. So, the description of the so-called fifth and sixth introduction events were inappropriate.

**Answer from author:**

We fully agree to the editor. The data analysis was subject to edition according to the recommendation and the corrections were included in the new version of the manuscript (Lines 285-288 and 322-362) and in the new figure 5 too.

8. In the phylogeographic analyses, the sequences from other continents (e.g. Asia, Africa) should be included.

**Answer from author:**

We agree to the reviewer. New sequences from other continents were added, the results were changed (Lines 285-362) to the dataset corresponding to Figure 4 and 5.

**Reviewer 1:**

In this paper authors track the migration of the dengue strain DENV in Central America, especially in the Columbia – Venezuela border. The paper is well-written. I am not expert in the phylogenetics approaches, but with whatever knowledge I have, I think methods are well done. Results are presented well, but I did not understand, why there is suddenly figure legend in the result text, I hope, this legend will go below the figure in the final version.

**Answer from author:**

We thank the reviewer for him/her words and agree to the last comment. However, this is part of the PLOS Template.

Also, I have suggestion to give. In the discussion, I was lost in reading how the dengue strain travelled through Columbia, Venezuela and other parts of Central America. I was thinking if this information can be depicted through a map, it will be beneficial to readers. Specially for people who are not familiar to the Central America geography.

**Answer from author:**

We agree to the reviewer. According to this recommendation, we added a new map in the part A of figure 1 (Lines 199-201).

**Reviewer 2:**

The authors conduct a phylogenetic analysis of DENV strains circulating in the border of Colombia and Venezuela. The results of this study provide some support for DENV cross-border transmission and suggests that border surveillance and characterization of imported and exported strains are very important. However, this study has many limitations.

1. The number of sequences included in the study was too small to illuminate the genetic characteristics of the border-prevalent strains. So the conclusions, geographical proximity between Colombia and Venezuela is favourable for the export and import of different cannot be strongly supported.

**Answer from author:**

We partially agree to the reviewer. We discuss the size of the sample as a limitation; however, the inclusion of several sequences previously reported in this country’s trough the las years, strongly support the conclusions. We included some sentences in the Discussion to clarify this item (Lines 501-504).

2. Is there more Asian strains included for the evolutionary rate calculating? Too few sequences will lead to bias in the calculation results. There are a large number of dengue sequences from Asia in the database.

**Answer from author:**

We agree to the reviewer. We included new sequences from different continents into the dataset to clarify the results (Lines 285-288) and the figures 4 and 5 were changed.

3. It will be better to annotate the meanings of the different colors in Figure 2 and Figure 3.

**Answer from author:**

We totally agree to the reviewer. The figure legends (Figure 2, lines 237-239 and Figure 3, lines 264-265) where modified according to the recommendation.
