# Supplementary material for: Phylogenetic and evolutionary analysis of dengue virus serotypes circulating at the Colombian–Venezuelan border during 2015–2016 and 2018–2019
Source: PLoS One. 2021 May 28;16(5):e0252379. doi: 10.1371/journal.pone.0252379 (PMC8162668; doi:10.1371/journal.pone.0252379)
Supplement: Attachment — Submitted filename: Response to reviewers (05-13-2021).docx [file pone.0252379.s004.docx]

**RESPONSE TO EDITOR COMMENTS (PONE-D-20-27563R1)**

Phylogenetic and evolutionary analysis of Dengue Virus serotypes circulating at the Colombian–Venezuelan border during 2015–2019

Marlen Yelitza Carrillo-Hernández, Julian Ruiz-Saenz, Lucy Jaimes Villamizar, Sara Maria Robledo Restrepo, Marlen Martínez-Gutierrez

**Editor Comments**

1. Please carefully check the Figures 4 and 5. Do the authors sure that the sampling year of the latest samples (the terminal braches in the MCC trees) were about 2025? Therefore, all tMRCA of the key nodes were wrong. In addition, please carefully determine which nodes can reflect the tMRCA of the strains from Colombia.

**Answer from author:**

We apologies for the mistakes in the figures. According to the revision we corrected the terminal branches un the MCC trees (Figure 4 and 5). Moreover, all tMRCA of the key nodes were corrected. Finally, all the corrections were included in the new version of the manuscript (Lines 311, 312, 320, 325, 326, 344, 345, 349, 351, 352, 357, 358 and 362

2. **Please clearly label each introduction event (at corresponding node).**

**Answer from author:**

We checked and corrected each introduction event in the new figures 4 and 5.

**3. Line141：previously reported primers: please provide refs.**

**Answer from author:**

We provided the reference to the end of the sentence (Reference number 19).

**4. Line 315: sequences from Santander reported during 2010–2016) and Venezuela (2005–2015): please check this sentence.**

**Answer from author:**

The sentence was corrected in the lines 314 to 316.
